# Editorial: Psychological Factors as Determinants of Medical Conditions, Volume II

**DOI:** 10.3389/fpsyg.2022.865235

**Published:** 2022-03-21

**Authors:** Andrea Caputo, Carmelo Mario Vicario, Valentina Cazzato, Gabriella Martino

**Affiliations:** ^1^Department of Dynamic and Clinical Psychology, and Health Studies, University of Rome “Sapienza”, Rome, Italy; ^2^Department of Cognitive Sciences, Psychology, Education and Cultural Studies, University of Messina, Messina, Italy; ^3^School of Natural Sciences and Psychology, John Moores University, Liverpool, United Kingdom; ^4^Department of Clinical and Experimental Medicine, University of Messina, Messina, Italy

**Keywords:** psychological factors, adherence, compliance, anxiety, stress, quality of life, emotional distress, chronic diseases

Life expectancy is increasing world-wide, thus age-related diseases are becoming a major health concern. Chronic diseases and related outcomes, such as osteoporosis and associated fractures, diabetes, endocrine, and cardiovascular disease, may seriously impact people's quality of life and their perceived quality of life (Guicciardi, 2017; Marchetti et al., 2017; Catalano et al., 2020; Vita et al., 2020; Di Bari et al., 2021; Martino et al., 2021a,b,c). This impact may, in turn, lead to psychopathological consequences (Di Giuseppe et al., 2020a; Giusti et al., 2021). Indeed, psychopathological symptoms frequently occur in tandem with chronic medical conditions and can even predict, and impact, mortality independently of a wide range of potential confounders (Kiecolt-Glaser et al., 2002; Lapolla et al., 2012; Kelly et al., 2019).

Moreover, psychological aspects may also drive individual behavior, including adherence to medical advice, deeply conditioning the management of chronic diseases (Marchini et al., 2020, 2021a,b). Anxiety and depression, emotional distress (e.g., alexithymia, abnormal disgust processing), body integrity identity disorders, cognitive deficits, lead to a variety of functional somatic disorders and affect a patient's attitude to treatment, which can impact their perceived quality of life (Vicario, 2013; Craparo et al., 2016; Di Giuseppe et al., 2019; Conversano et al., 2020; Martino et al., 2020; Barchetta et al., 2021; Gangemi et al., 2021; La Rosa et al., 2021; Liotta et al., 2021; Vicario et al., 2021; Martino et al.). Therefore, overturning the usual causal direction body-mind, evidence exists regarding the key role of psychopathological factors in the history of chronic illness (Castelnuovo, 2010; Castelnuovo et al., 2015; Conversano, 2019; Martino et al., 2019; Caputo, 2020; Vicario et al., 2020b). A strict evaluation of the psychological variables could contribute to a better understanding of the individual condition and possibly predict the risk of the onset of new medical diseases or complications (Salehinejad et al., 2020; Lucifora et al., 2021; Sardella et al., 2021). This could suggest a new direction in psychopathological research and prevention, leading to screening subjects at risk for medical events in order to individualize and improve diagnostic and therapeutic approaches (Vicario and Nitsche, 2013; Di Giuseppe et al., 2020b,c).

Following the previous Research Topic on Psychological Factors as Determinants of Medical Conditions (Martino et al., 2019), in this second volume, we aim to update the latest developments including interdisciplinary and multidisciplinary contributions in order to understand the interrelations among psychopathological aspects, somatic symptoms, and medical outcomes.

Our collection includes 17 articles—four opinion articles, five review studies, and eight research studies—which address several aspects of body-psyche integration from a theoretical, research and intervention perspective. Nine articles focused on clinical samples, with specific regard to developmental psychopathologies (Di Giuseppe et al.; Sorrenti and Filippello), neurological disorders (Kelly et al.), learning disabilities (Cataudella et al.), personality disorders (Eikeseth et al.; Williams et al.), and chronic and medical conditions (Conversano and Di Giuseppe; Guicciardi et al., Martino et al.). Whereas, eight articles dealt with general populations in order to examine the role of personality characteristics or dispositions (Conversano et al.; Conversano et al.; Merlo et al.; Rymarczyk et al.) and some variables connected with emotion processing (Conversano; Gaggero et al.; Di Giuseppe et al.; Di Giuseppe et al.). Overall, this collection contributes to deepening the interrelations between physical and mental health and well-being, highlighting the need to summarize the existing research knowledge (Cataudella et al.; Conversano et al.; Gaggero et al.; Kelly et al.; Martino et al.), provide new empirical evidence (Conversano et al.; Di Giuseppe et al.; Guicciardi et al.; Merlo et al., Williams et al.), develop and validate measurement tools (Di Giuseppe et al.; Eikeseth et al.; Rymarczyk et al.), and discuss the advances in clinical treatments and approaches (Conversano; Conversano and Di Giuseppe; Di Giuseppe et al.; Sorrenti and Filippello).

We hope that the articles here presented may provide interesting insights and help researchers and practitioners grasping the complexity of human mind and embracing a health promotion perspective, attentive to improving individuals' quality of life and adjustment processes at a physical, mental, and social level (Tomai et al., 2019; Vicario et al., 2020a; Conversano et al.; Conversano et al.).

It has been very pleasant and exciting for us to continue dealing with such issues and be newly involved in this Research Topic. In this regard, our thanks go out to all of them who made it possible, specifically Authors for their endeavors and interesting contributions, Reviewers for their competences and the time devoted, and the entire Frontiers Editorial and Developmental Staff for their patience and precious continued assistance.

## Author Contributions

AC and GM wrote the first draft of the manuscript and revised it critically. CMV and VC provided opinions on it. All authors read and approved the submitted version.

## Conflict of Interest

The authors declare that the research was conducted in the absence of any commercial or financial relationships that could be construed as a potential conflict of interest.

## Publisher's Note

All claims expressed in this article are solely those of the authors and do not necessarily represent those of their affiliated organizations, or those of the publisher, the editors and the reviewers. Any product that may be evaluated in this article, or claim that may be made by its manufacturer, is not guaranteed or endorsed by the publisher.
